# Measuring change in biological communities: multivariate analysis approaches for temporal datasets with low sample size

**DOI:** 10.7717/peerj.11096

**Published:** 2021-04-08

**Authors:** Hannah L. Buckley, Nicola J. Day, Bradley S. Case, Gavin Lear

**Affiliations:** 1 School of Science, Auckland University of Technology, Auckland, New Zealand; 2 School of Biological Sciences, Victoria University of Wellington, Wellington, New Zealand; 3 School of Biological Sciences, University of Auckland, Auckland, New Zealand

**Keywords:** Beta diversity, Community variation, Biodiversity, Compositional change, Multivariate analysis, Species turnover, Temporal change, Zeta diversity, Temporal variability, Time series

## Abstract

Effective and robust ways to describe, quantify, analyse, and test for change in the structure of biological communities over time are essential if ecological research is to contribute substantively towards understanding and managing responses to ongoing environmental changes. Structural changes reflect population dynamics, changes in biomass and relative abundances of taxa, and colonisation and extinction events observed in samples collected through time. Most previous studies of temporal changes in the multivariate datasets that characterise biological communities are based on short time series that are not amenable to data-hungry methods such as multivariate generalised linear models. Here, we present a roadmap for the analysis of temporal change in short-time-series, multivariate, ecological datasets. We discuss appropriate methods and important considerations for using them such as sample size, assumptions, and statistical power. We illustrate these methods with four case-studies analysed using the R data analysis environment.

## Introduction

A key task for ecologists is to monitor, quantify, analyse, and predict temporal changes in the structure of ecological communities as a function of ongoing changes in climate, land use, and other environmental drivers. The structure of communities—the identities (‘composition’), number (‘richness’) and relative abundances (‘evenness’) of taxa—changes through time because of fluctuations of individual populations and local colonisations and extinctions (Micheli et al., 1999; Legendre & Gauthier, 2014). These temporal dynamics are governed by both intrinsic factors, including intraspecific and interspecific interactions, colonisations and extinctions, and extrinsic factors, which are processes that relate to periodic disturbances and changing environmental conditions, for example shifts in temperature or drought (Connell & Slatyer, 1977; Brown & Lawson, 2010). Understanding the temporal dynamics of community structure can illuminate fundamental ecological processes, including effects of individual life histories on rates of ecosystem change, the relative importance of biotic and abiotic factors in determining community structure, or how taxa and the networks in which they are embedded respond to environmental change (Tilman, 1999; Hobbs, Yates & Mooney, 2007; Kampichler et al., 2012). Temporal changes in taxon richness and composition can also provide both simple summaries of ecosystem change and information that can be translated rapidly into action, either to protect and restore declining taxa, or to staunch unsustainable growth of unwanted organisms (Alday et al., 2013).

Multi-taxon, multivariate datasets can be more sensitive to changes in explanatory or predictor variables (e.g. pH, temperature, soil type), than univariate summary statistics, such as taxon richness, diversity, or biomass (Micheli et al., 1999; Chao et al., 2014), and hence more informative about causal processes (Collins et al., 2008). However, quantitative methods for investigating temporal dynamics of multivariate measures of community structure (henceforth ‘community dynamics’) are more complex than methods used for univariate data (Schaefer, Gido & Smith, 2005; Legendre & Legendre, 2012). Further, methods for the analysis of spatial multivariate community structure data (Whittaker, 1960; Legendre & Legendre, 2012) have received far more attention to date than methods for analysis of community dynamics data. Well-known concepts used to describe and analyse spatial changes in community structure—for example nestedness, beta diversity, taxonomic turnover—have been transferred to the temporal domain (Azeria & Kolasa, 2008; Anderson et al., 2011; Legendre & Gauthier, 2014), and some well-developed multivariate methods for large spatial datasets have been used to detect community dynamics (Legendre & Gauthier, 2014). Yet, comparisons of multivariate ecological data through time have additional, non-trivial challenges, such as the difficulty of distinguishing spatial variation in community composition from temporal variation, which can manifest in a wide range of forms including directional, cyclical and stochastic (Philippi, Dixon & Taylor, 1998; Micheli et al., 1999; Collins, Micheli & Hartt, 2000; Schaefer, Gido & Smith, 2005).

A recent quantitative review of the temporal dynamics literature (Buckley et al., 2021) showed that 75% of the 548 studies reviewed used, on average, fewer than 16 temporal replicates (median = 7) in their analyses. Further, results indicated that researchers have primarily relied on descriptive analyses to interpret patterns of community change, by presenting changes in taxon presence or abundance in table or figure format, or by using ordination-based methods. Augmenting such descriptions with methods that quantify temporal dynamics in taxonomic composition in more powerful and specific ways, allows more quantitative and comparable descriptions of spatiotemporal patterns and explicit hypothesis-testing of underlying mechanisms. There have been many promising developments over recent decades in sophisticated methods for analysing multivariate datasets (Angeler, Drakare & Johnson, 2011; Holmes, Ward & Wills, 2012; Legendre & Gauthier, 2014). However, these methods can often be challenging to apply to short time series and/or datasets with very large numbers of taxa compared to samples, such as datasets typically generated using DNA-based methods like next-generation sequencing. Therefore, we provide here descriptions of, and recommendations for, a range of quantitative multivariate methods to assess temporal changes in species communities, specifically for typical situations where there are few temporal replicates in a statistical sense (‘small sample size’, Jenkins & Quintana-Ascencio, 2020), that is fewer than 25 temporal replicates: We provide guidance for approaching such multivariate analyses as a four-step process: Step 1: Data exploration, Step 2: Selecting multivariate methods, Step 3: Testing for statistical significance, and Step 4: Reporting on the analysis workflow. We suggest this four-step procedure will be particularly beneficial for researchers who are undertaking analyses of multivariate temporal community dynamics data for the first time. Finally, we provide four worked case study examples with R code (Codes S1–S4). Specific terms used in this article are listed in a glossary (Table 1).

**Table 1:** Glossary of terms used in this article.

Term	Definition
Beta diversity	How taxonomic composition changes across a set of samples. The variation in taxonomic composition among samples. Often used to refer to compositional dissimilarity
Community	Assemblage of multiple populations of different taxa defined by the researcher
Community composition	Identities and abundances of taxa in a biological community. Synonymous with taxonomic composition
Compositional overlap	A list of taxa that are shared between two samples
Convergence	Taxonomic composition of two or more samples becomes more similar in a predictable way
Cyclical dynamics or periodicity	Taxonomic composition diverges and then re-converges on the original composition repeatedly over time
Dissimilarity index (distance measure)	Numeric measure of ecological resemblance between two samples based on taxonomic composition. When two samples have similar taxonomic composition then their dissimilarity is high. Often synonymous with dissimilarity measure and dissimilarity coefficient. Can be converted to similarity index
Metric dissimilarity index	A type of dissimilarity that takes the minimum value of zero when two samples are identical. Symmetric in that the dissimilarity between samples A and B is the same as the dissimilarity between B and A. Fulfills the triangle inequality axiom, which states that for three samples, the distance between two of them cannot be larger than the sum of the other two
Non-metric dissimilarity index	Does not fulfil one or more properties of metric dissimilarity indices
Dissimilarity matrix	A matrix of dissimilarity indices for multiple samples
Distance index	A subset of a type of dissimilarity index
Divergence	Taxonomic composition of two or more samples becomes less similar in a predictable way over time
Homogenisation	Taxonomic composition of a set of samples becomes more similar, often with a concurrent decrease in diversity
Resilience	Significant change in composition, reverting back to the original state over time
Succession	Individual or groups of taxa are replaced in a predictable sequence
Synchronicity	Different groups of taxa change in similar ways at the same time. These groups may be functional or taxonomic
Taxa richness	The number of taxa in a sample
Taxa diversity	The number of taxa in a sample including information on their relative abundances and evenness across samples
Taxa turnover	Rate of change in taxonomic composition over time

### Step 1: data exploration

As recommended for the analysis of any ecological datasets (Zuur, Ieno & Elphick, 2010), exploratory analyses can be essential for pinpointing the most appropriate analysis methods for a given dataset. We therefore suggest conducting exploratory data analyses (sensu Zuur, Ieno & Elphick, 2010) in advance of determining the analysis pathway. Here we outline several recommended procedures to assess the data structure in ways that can assist getting familiar with the dataset, preparing for and selecting the appropriate analysis methods, and interpreting the results (Table 2). This is particularly important where users are dealing with datasets that they have inherited from other researchers.

**Table 2:** Descriptions of the outputs obtained from analysis methods for temporal change in community composition with examples from the literature.

Method (text section, R code)	Description of outputs	Key article(s) using each method
Raw dissimilarity values (Method 1.1; Code S3)	Various, depending on the method. For example, the dissimilarity-overlap method results in a graph of pairwise dissimilarity values against the proportion of individuals that taxonomically overlap; overlaid on this is a non-parametric, non-linear regression line, the slope of which indicates whether or not samples are changing in similar ways or not; this is compared to a null hypothesis of zero slope.	Bashan et al. (2016)
Mean dissimilarity (Method 1.1; Code S3)	Mean dissimilarity values for data subsets. Values can be used in subsequent graphs, analyses or maps.	Collins (1992)
Mean dissimilarity as response in regression (Method 1.1; Code S2)	Regression statistics indicating the significance of the relationship between mean dissimilarity and explanatory variables, which includes time if dissimilarity is calculated for different spatial samples, or will only include change in non-time explanatory variables, if dissimilarity is calculated across time.	Collins (1992); Barros et al. (2014)
Beta or zeta diversity time-lag graph (Methods 1.2 & 4; Code S4)	Graph showing the relationship between temporal diversity and time lag (distance in time). In the case of zeta and multi-site dissimilarity, time-lag graphs are specific to each number of samples in the subset (e.g. zeta order). See full description of zeta diversity below.	Hui & McGeoch (2014), Mcgeoch et al. (2017)
Beta or zeta time lag regression (Methods 1.2 & 4; Code S4)	Regression statistics indicating the significance of the relationship between temporal diversity and time lag (distance in time). In the case of zeta and multi-site dissimilarity, time-lag analyses are specific to each number of samples in the subset (e.g. zeta order).	Mcgeoch et al. (2017)
Beta diversity decomposition (nestedness, turnover) (Method 1.3, Code S2)	Beta diversity values for nestedness and turnover that can be used in subsequent graphs (e.g. time lag) or analyses of explanatory variables.	Baselga & Orme (2012)
Beta diversity decomposition (SCBD: species contributions to beta diversity; LCBD: local contributions to beta diversity) (Method 1.3; Code S2)	List of relative contributions of taxa to the overall compositional variation. Matrix of LCBD values showing the relative contribution of each sample-time combination to the overall compositional variation. Values can be used in subsequent graphs or analyses of explanatory variables.	Legendre & De Cáceres (2013), Legendre & Gauthier (2014), Lamy et al. (2015)
Ordination (constrained) (Method 1.4)	Ordination diagram and table of statistics (see the text section ‘Ordination’ for more details and other possible outputs).	Anderson & Willis (2003), http://ecology.msu.montana.edu/labdsv/R/
Ordination site scores in general linear model or non-linear regression (Method 1.4; Code S2)	Regression statistics indicating the significance of the relationship between species composition and explanatory variables.	Day & Buckley (2013)
Temporal coherence (Method 1.4; Code S2)	A single value representing the degree of consistency in temporal change among samples for the dataset or subset considered (see the text section ‘Ordination’ for more details).	Rusak et al. (1999), Angeler & Johnson (2012)
Principal response curves (Method 1.4; Code S2)	Principal response curve diagram comparing trajectories in compositional space of different sites or samples over time relative to the baseline or control.	Auber et al. (2017)
Pivot days (Method 1.6; Code S2)	A list of time points is identified at which significant shifts in composition have occurred.	Lellouch et al. (2014)
Community trajectory analysis (Method 1.7)	Principal coordinates analysis diagram with trajectories showing change in species composition of samples through time. This is paired with diagrams showing the overall dissimilarity in complete trajectories between samples. Trajectory statistics, including shape, size and direction are also obtained.	De Cáceres et al. (2019)
Rank abundance distributions (Method 2; Code S4)	Species ranked from highest to lowest relative abundance on graph. Can be combined with dissimilarity methods and rank clocks.	Avolio et al. (2015), Hallett et al. (2016)
Venn diagrams, overlap and time cores* (Method 3; Code S2)	Venn diagram showing the number of taxa shared and unshared between two or more subsets of samples. Lists of shared and unshared taxa. Time cores results generate lists of taxa that occur consistently through time for a set of samples. Specifically useful for detecting taxon replacement over time	Vallès et al. (2014), O’Sullivan et al. (2015)
Zeta diversity (Method 4; Codes S3 & S4)	Zeta diversity values for each zeta order (number of samples in subsets) that can be used in subsequent graphs (e.g. zeta diversity vs. order) or analyses of explanatory variables	Hui & McGeoch (2014), McGeoch et al. (2019)
Synchrony (Method 5; Code S4)	A single value representing the degree to which taxa are changing in a similar way over time, calculated for a given sample by species matrix across a set of times. Can be compared for different time windows or different subsets of samples (e.g. experimental treatments)	Loreau & De Mazancourt (2008), Gouhier & Guichard (2014)
Turnover rates (Method 6; Code S3)	Percent change in species composition between time points	Diamond (1969)
Joint species distribution models (Method 7)	Observed vs. predicted patterns in taxon associations. Can test spatially and temporally-specific scenarios or hypotheses by modelling taxon associations in relation to environmental variables. Traits and phylogenetic information can be included. Presentation of results depends on the hypothesis of interest, for example predicted and observed changes in taxon communities can be mapped along environmental gradients	Ovaskainen et al. (2017a, 2017b)

**Note:**

We provide a reference to the relevant text section within Step 2 and the location of example R code in supplementary material (Codes S1–S4). Where R code is not listed as available in supplementary material, it is available in the article cited for the method. LCBD refers to ‘Local Contributions to Beta Diversity’ and SCBD refers to ‘Species Contributions to Beta Diversity’.

#### Count the number of taxa, the number of samples and the number of empty samples and missing data points

As some multivariate methods are more susceptible than others to the influence of rare taxa, the consideration of the ratio of the number of taxa to the number of samples in a dataset is an important initial exploratory step (Lepš & Šmilauer, 2003). It is common for long-term datasets to contain missing values; these values should be denoted as missing (i.e. ‘NA’ in R) and not given zero or other numerical values. The importance of missing values and how to deal with them during data analysis depend on the number of missing data points and how they are distributed across the dataset. Multivariate analyses based on certain compositional dissimilarity measures cannot be applied to datasets containing any samples where no taxa are present (McCune & Grace, 2002; Legendre & Legendre, 2012). Many distance measures cannot be computed if there are empty samples or missing values. Deleting entire rows or estimating missing data so that the dataset can be analysed may be an option in some cases (McCune & Grace, 2002). However, other measures, such as the Gower coefficient, can be calculated while ignoring comparisons among samples with missing data (Legendre & Legendre, 2012; coefficient S15: 278). Comparing results based on presence-absence data with those generated using relative abundance data allows us to evaluate the role of taxon abundances in generating temporal patterns (Angeler & Johnson, 2012). In contrast, empty samples may be important for analyses where we are interested in understanding responses to disturbance and local extinctions.

#### Ensure that relative abundances are comparable

Estimating abundances of each population within the community has its own important considerations, such as variation in taxon detection probabilities, which have been addressed elsewhere (e.g. animals: Gaston & McArdle, 1994; amplicon data: Amend, Seifert & Bruns, 2010). However, once the taxon-sample matrix has been collated, abundance data from different taxa can be compared. If abundances were measured on different scales, the data should be standardised or transformed prior to analysis, for example in the case of aquatic communities, macroinvertebrate abundances are likely to have been estimated using different volumes of water to macroinvertebrates (Legendre & Legendre, 2012). In such cases, taxon abundance values within each sample can be divided by the total abundance within the sample, or can be standardised across all samples so that each taxon has a mean of zero and standard deviation of one (Borcard, Gillet & Legendre, 2011). Alternatively, a distance measure that incorporates a standardisation can be used, for example, Hellinger distance (Legendre & Gallagher, 2001; Anderson et al., 2011; Legendre & Legendre, 2012).

#### Examine taxon frequency distributions and response curves

Taxon frequency distributions can be visualised by plotting a histogram for each taxon of the number of individuals or their relative abundance across samples. This is important as some multivariate analyses rely on the assumption that these frequency distributions take particular shapes such as normal distribution or a linear response across an environmental gradient that samples were collected from; for example, canonical correspondence analysis (CCA) assumes that taxa have optima along environmental gradients (‘unimodal’ responses). Examination of these distributions can reveal whether taxa are responding strongly to environmental gradients in the dataset and whether the dataset conforms to analysis assumptions. Data transformations, such as log or square-root, can be applied to normalise abundance data, if required.

#### Determine the ratio of temporal to spatial variation in composition

The amount of spatial and temporal replication is important in determining the method of analysis because few samples with many measurements over time would be analysed differently from many samples measured only a few times. The ratio of the number of independent replicates to the number of explanatory variables is also important for temporal analyses because it affects the degrees of freedom available for statistical testing (Johnstone & Titterington, 2009). If the data have a nested structure this needs to be accounted for in the analysis, particularly if one wants to report significance values. For example if plant species were recorded in quadrats that were nested within transects that were nested within sites, then quadrats are not fully independent replicates. Similarly, information from technical replicates (such as PCR products) should be kept separate at this stage because they provide useful information about variability at different scales in the dataset. Thus, it is inadvisable to average or pool data until the significant sources of variation are understood. For instance, if there is a high degree of spatial variation among samples in the dataset, this can alter analysis choices or how results are presented and interpreted (Thrush et al., 2008). The relative amount of change in taxonomic composition can be estimated from the differences in sample scores on detrended correspondence analysis (DCA) ordination axes, for example, the ‘gradient length’ statistic quantifies the turnover in taxonomic composition (Lepš & Šmilauer, 2003).

### Step 2: selecting multivariate methods

Here we describe a range of methods to assess community dynamics in small sample size datasets (fewer than 25 temporal replicates): (1) pairwise dissimilarity-based methods, (2) rank abundance distributions, (3) Venn diagrams and time cores, (4) zeta diversity, (5) synchrony, (6) turnover rates, and (7) joint species distribution models. The selection of an appropriate analytical method also can be influenced by the kind of output desired (Table 3) or how patterns will be visualised. For instance, some analyses result in outputs that require more effort to interpret than others, whilst others show differences in taxon responses or generate outputs that can be added to a geographic map. Where necessary, R code is provided to demonstrate how to implement analyses (Codes S1–S4).

**Table 3:** Data visualisation methods and considerations for their use in temporal community dynamics studies.

Methods	Description and example references	Considerations
Time lag graphs (Method 1.2; Fig. 3; Code S1)	Pairwise dissimilarities among all times (for each site) combinations are plotted against the temporal lag (distance in time) to display the relative change in composition over different periods of time (Collins, 2000; Collins, Micheli & Hartt, 2000)	Can be used to visualise the majority of temporal patterns and works well for both smaller or larger numbers of temporal replicates (at least five samples)
Ordination axis sample scores (Method 1.4; Fig. 1; Code S1)	Symbols (Noll et al., 2005), envelopes (Rayner, Pusey & Pearson, 2009), or trajectories (Rojas-Sandoval, Meléndez-Ackerman & Fernández, 2012) can be added to ordination diagrams to display the relative compositional change among temporal samples	Can be used to show convergence, divergence, or in/stability of sets of samples. Useful for many samples and taxa. Ordination sample scores can also be used in other graphs or maps to display spatiotemporal compositional variation. PRC is a good way to visualise change relative to a control
Relative abundance distributions (Method 2; Code S4)	Plot the relative abundance of all taxa in the community (Lambshead, Platt & Shaw, 1983; Stead, Schmid-Araya & Hildrew, 2003; Ruhl, 2008)	Shows changes in evenness over time where separate relative abundance distributions are plotted for each time. Useful only for small numbers of comparisons, for example two or three sampling times
Venn diagrams (Method 3; Code S2)	Displays the overlap in species composition among samples (O’Sullivan et al., 2015)	Suitable for visualising taxon replacement while specifically identifying the taxa driving compositional shifts. Useful only for small numbers of temporal replicates
Time cores (Method 3; Code S2)	Identify ‘core’ groups of taxa that remain present over time (Vallès et al., 2014). This can be done by calculating the overlap in composition between samples	Useful for visualising synchronicity in compositional shifts
Heat maps (Fig. 7; Code S3)	A grid representing spatial and/or temporal samples where cells are coloured by a gradient representing relative abundances, ordination sample scores, or compositional change (Pedersen et al., 2017; Pereira et al., 2017)	Useful for representing change between time points across sample space, even in the case of complex patterns
Geographic maps	Grid cells or other geographic polygons displaying numeric values representing relative abundances, ordination sample scores, or compositional change (Clavero & García-Berthou, 2006)	Useful for representing change between time points where variation in geographic space is also important. Useful for relatively few time points
Abundance graphs	Bar graphs, boxplots, scatterplots, or pie charts showing relative abundance of different taxa at different times (Xiong et al., 2014; Chapman & McEwan, 2016; França et al., 2016)	Can show complex temporal dynamics such as non-linearities in species’ responses and synchronicity among different taxa, but useful only for a small number of taxa
Relative abundance tables	Relative abundance or percent dominance values across time (Shertzer, Williams & Taylor, 2009)	Useful only for a small number of taxa and spatiotemporal samples

**Note:**

Relevant text sections, figures, and example R code are referred to. If visualisations are not presented, they are available in the references listed.

#### Method 1: pairwise dissimilarity-based approaches

Pairwise dissimilarity methods are the most common way biologists quantify the difference in the identities of taxa occurring in two samples (Buckley et al., 2021). The result is a single number that measures the distance in compositional space between the two samples. Dissimilarity can also be represented as similarity, that is, 1—dissimilarity (Whittaker, 1960; Anderson et al., 2011; Magurran, 2013). When applied to multiple samples, these methods result in a matrix of all possible comparisons (a triangular dissimilarity matrix).

A community dataset may be an abundance matrix (number of individuals), relative abundance matrix (e.g. percent cover, sequence abundances), rank-abundance matrix (ordinal abundance ranks), or a taxon-occurrence (presence-absence) matrix. These different response variables can influence the choice of analysis methods, including which dissimilarity measure to use, so it is important to explore different metrics to determine which method gives the best representation of differences in composition. For instance, the commonly-used Euclidean distance measure is not usually appropriate for taxon abundance data, which are often characterised by non-linear relationships among taxa, abundance values measured on different scales, or by the ‘double-zero problem’, which is where samples that are missing the same taxa have lower dissimilarity (Borcard, Gillet & Legendre, 2011).

Different authors often use different names for the same dissimilarity measure, so we recommend Legendre & Legendre (2012 Table 7.4: 324) for a comprehensive and consistent guide. However, other definitive sources for overviewing and comparing dissimilarity indices are provided by Anderson et al. (2011), Legendre & De Cáceres (2013), and Magurran (2013). The development of dissimilarity indices is an active area of research, including those specifically applicable to temporal data (Podani & Schmera, 2011; Baselga, 2012; Shimadzu, Dornelas & Magurran, 2015) and some contributions aid in improving dissimilarity measures for use in certain circumstances, such as imperfect detection of taxa (Chao et al., 2006) or where phylogenetic information is available from sequencing that has been used to determine microbial taxa in samples (unifrac; Lozupone & Knight, 2005; Lozupone et al., 2007).

##### Method 1.1 using the raw dissimilarity values

Raw dissimilarity values for samples are typically used in one of two ways to assess changes in community composition. First, compositional dissimilarity can be calculated among samples taken at different locations to obtain a measure of spatial heterogeneity in community composition (Code S3). To use these values to analyse temporal community dynamics, pairwise dissimilarity (spatial dissimilarity) is calculated separately for each sampling time and the means of these spatial dissimilarity values are then compared across time visually in a table (Collins, 1992) or graph, or used in a correlation or regression analysis, where time is the predictor variable and amount of spatial variability at each sample/site is the response (Barros et al., 2014). Dividing mean dissimilarity by the number of taxa can generate an independent measure of compositional change (Cleland et al., 2013). Such comparisons over time can be done separately for different groups of samples, such as different experimental treatments, to test for predictors of differences in temporal change in composition. Bagchi et al. (2017) illustrate predictions of how temporal differences in dissimilarity can include non-linear patterns and how linear and non-linear regression modelling can be applied to pairwise dissimilarity values to assess drivers of temporal dynamics.

Second, if a set of samples were all taken at the same site at different times, ‘temporal heterogeneity’ can be calculated using the mean dissimilarity of the entire sequence of temporal samples to obtain a single measure of within-site temporal variability (Collins & Smith, 2006). If multiple sites are sampled through time, this method can be applied at different spatial scales (Collins, 1992; Collins & Xia, 2015) to examine spatial scale-dependent temporal dynamics (Collins & Smith, 2006). Regression approaches that relate mean temporal dissimilarity values to other explanatory variables for sites or samples (Code S2) could be used to explore spatial variability in temporal change (Collins, 1992). Alternatively, the ‘dissimilarity-overlap’ method (Bashan et al., 2016; Kalyuzhny & Shnerb, 2017) can be applied to raw dissimilarity values to test for similarity among samples in community dynamics. This method uses the relationship between the pairwise dissimilarity values and the proportion of individuals in pairs of samples that overlap taxonomically to detect if changes in taxonomic composition (degree of overlap) alters relative abundances in a consistent way across samples (dissimilarities). A recent development shows that for a given sample, changes in composition can be partitioned into whether the change is driven more by gains or losses of species (Legendre, 2019). We provide a worked example of the use of raw dissimilarity data in temporal community dynamics analysis in the Code S3 supplement.

##### Method 1.2 time-lag analysis

A time-lag analysis involves relating the amount change in composition to the amount of change in time across increasing temporal distances, called ‘lags’ (Table 2; Code S4). For example, Collins & Xia (2015) compared plant species composition of repeatedly-measured grassland transects across all time lags between 1989 and 2008 (from a 1- to 19-year difference). There are two ways of assessing the time-lag effect on community dissimilarity: graphically (‘time-decay curve’) and statistically (time-lag regression analysis). For meaningful inference, time-lag graphs require at least five samples in time and statistical time-lag analysis requires at least ten samples for each temporal sequence. For both approaches, the pairwise compositional dissimilarity (temporal beta diversity) among temporal samples is calculated first.

To generate a time-decay curve, pairwise temporal dissimilarity values are plotted against temporal distance between samples to show how community dissimilarity changes as samples become further apart in time (Code S4; Collins, 2000; Collins, Micheli & Hartt, 2000; Hallett et al., 2016). This graph can be then used to visually assess whether community composition is converging (becoming more similar) or diverging (becoming less similar) over time (Collins, 2000). For example, Bar-Massada & Hadar (2017) plotted temporal beta diversity (as Bray–Curtis dissimilarity) of plants in grazed and ungrazed treatments in Mediterranean herb communities, revealing that composition diverged over time, regardless of grazing treatment. Alternatively, temporal community dissimilarity can be graphed against dissimilarity in environmental variables (rather than time), if the primary research focus is on response of the community to environmental change rather than time per se.

Time-lag regression analysis is appropriate for datasets with greater than ten time points and can be used to assess directional, cyclic or stochastic changes within communities (Collins, 2000; Collins, Micheli & Hartt, 2000). For time-lag regression, the pairwise compositional distances are plotted against the square-root of the time lag, which reduces the bias from having fewer data points at larger time lags (Code S4; Collins, 2000; Collins, Micheli & Hartt, 2000). A regression line is then fitted to determine the pattern of compositional change. Given that the number of points in the regression is the number of sample pairs, and thus pseudoreplicated, the focus of interpretation should be on the shape and slope of the pattern, rather than the significance of the regression line, particularly when comparing regression lines among different sites or communities. A positive relationship indicates that compositional changes in communities is directional, because sampling points that are further apart in time are more dissimilar than those that are closer together in time (i.e. Fig. 2 in Collins (2000)). A negative slope shows that some convergence towards the taxonomic composition at one of the earlier time points has occurred, because measurements close together in time are more dissimilar in composition compared to those further apart in time. No slope implies stochastic changes in the community over time. Non-linear regressions also can be used to describe more complex temporal patterns (Magalhães et al., 2007). Time-lag regression analysis is appealing because it is intuitive, can be used to produce just one statistic (the regression coefficient), and can be easily visualised (e.g. freshwater macroinvertebrates: Bêche & Resh, 2007; gut bacteria: Dimitriu et al., 2013; plants: Collins & Xia, 2015). However, time-lag analysis only provides one value for each sampling unit, so further analyses may be required to investigate changes within treatments or groups.

##### Method 1.3 beta diversity decomposition

‘Beta diversity’ is a term that has been used for several different types of measures in ecology (Whittaker, 1960; Baselga, 2010; Legendre & De Cáceres, 2013; Legendre, 2019), including as a synonym for compositional dissimilarity. It describes how taxonomic composition changes across a set of samples. A number of recent advances in the calculation of beta diversity have illustrated that, if calculated in particular ways, it can be ‘partitioned’ into different components that relate to different predictions of how taxa or their abundances change in space or time (Baselga, 2010; Ellison, 2010; Legendre & De Cáceres, 2013). Beta diversity decomposition methods can be applied to many types of community data. The outputs are focussed on species replacement and whether samples are subsets of each other (Table 2), so detecting patterns such as convergence or divergence is relatively straightforward. However, depending on what subsets of the data are used in separate calculations, beta diversity decomposition is equally able to detect rapid shifts in composition, such as resulting from environmental perturbation, as well as increases or decreases in compositional variability over time.

Baselga (2010) and Baselga & Orme (2012) present a ‘nestedness’ approach to quantifying community dynamics based on beta diversity decomposition (Code S2). In this method, among-sample dissimilarity is first measured as Sørensen’s dissimilarity: (*b* + *c*)/(2*a* + *b* + *c*), where *a* is the number of shared presences and *b* and *c* are the number of unshared presences in each of the two samples. The Simpson’s dissimilarity index, also known as ‘species turnover’: *b*/(*b* + *a*), can then be subtracted from this value, resulting in a ‘nestedness’ measure. This nestedness measure reflects the degree to which the species composition of smaller samples (lower species richness) is encompassed by the species composition of larger samples (higher richness); samples may differ in space or time. In contrast, ‘species turnover’ measures the degree to which samples do not share species: how the identities of species differ among samples. It is useful to separate these two components (nestedness and turnover) to obtain a more informative quantification of beta diversity that reflects differences in the underlying processes. Such separation can be done either pairwise or for multiple sets of samples (Baselga, 2010, 2017), and has been used to decompose temporal beta diversity in tests of hypotheses unique to temporal data (Baselga & Orme, 2012).

In contrast, Legendre & De Cáceres (2013) defined beta diversity as the sums of squares in the variation of taxon abundance (or presence) corrected for the number of samples (Code S2). They partitioned this measure of beta diversity into two different components: ‘Species contribution to beta diversity’ (SCBD) and ‘local contribution to beta diversity’ (LCBD), which describe, respectively, the relative contributions of different taxa and samples to overall beta diversity (Legendre & Gauthier, 2014). This method allows for tests of predictions of patterns in temporal community dynamics related to different taxa in the dataset. For example, Lamy et al. (2015) decomposed beta diversity (based on Bray–Curtis dissimilarity) of fish communities at each of 13 reef sites into contributions from biomass replacement and biomass differences over time. They demonstrated that total biomass in the community changed little, but that the observed significant temporal changes in fish composition resulted from differential changes in the relative biomass of species. Spatiotemporal patterns in the two components of fish community beta diversity were visualised using principal coordinates analysis (PCoA). We provide a worked R code example for beta diversity decomposition in the Code S2 supplement.

##### Method 1.4 ordination

Ordination summarises multivariate community data by optimising relationships between high-dimensional samples and taxa in low-dimensional space to detect important ecological gradients in communities (Table 2). This can be an excellent way to summarise and visualise variation in a community dataset where samples are labelled to show spatial or temporal patterns. However, the dimensionality reduction means that, in comparison to raw dissimilarity methods, much information may be lost, so ordination methods are more useful for strong, simple patterns along gradients than for analysing complex changes in community composition over time, such as tipping points, cyclical patterns or synchronicity (different components of the community changing in the same ways at the same times). Further, ordinations have a tendency to distort the relative distances (dissimilarities) between samples, depending on, for example, differences in the number of rare taxa, and so values from distance-based indices may not be comparable, even in different parts of the same ordination. Ordination diagrams and their statistics computed for different matrices (different sets of taxa or sites) are not comparable; results of individual ordinations cannot be compared among them. The partial exception to this is Procrustes analysis, which can measure the distortion, or ‘lack of fit’ between two ordinations, or dissimilarity matrices (Jackson, 1995; Peres-Neto & Jackson, 2001). It can be used to identify which taxa are most important in differentiating between the two ordination patterns.

Ordination methods allow only weak inference of the relationship between taxa and community dynamics if the temporal pattern is complex relative to the ordination axes because some ordinations, such as DCA, only provide ordination axis scores for taxa; others do not provide ‘species scores’ at all, such as PCoA and NMDS (Codes S2 and S4). Where species scores are not provided, weighted averaging can be used to plot points for each taxon on the ordination diagrams to relate differences in sample composition to differences in taxon presence or relative abundance if the associations between samples and taxa are to be examined (McCune & Grace, 2002).

Despite the above limitations, ordinations are useful for analysing temporal community dynamics if we do more than simply plot the ordination diagram. For instance, linear models can be used regress ordination axis scores against time and other explanatory variables of interest (Code S4; Berthon et al., 2014; Broadway et al., 2015). Cotton et al. (2015) used DCA scores as a response variable in a linear mixed model to investigate the importance of carbon dioxide, ozone, and time on change in arbuscular mycorrhizal fungal community composition. This approach should be applied to axis scores generated by methods, such as PCoA and DCA, where the first axis represents the gradient explaining the greatest amount of variation in composition, followed by the second axis that explains the next greatest amount, and so on; this is not the case for one of the most popular methods, non-metric multidimensional scaling (NMDS; McCune & Grace, 2002). Therefore, it is recommended that NMDS scores are not used as response variables to model community dynamics unless the amount of variation explained by each axis is clearly stated in order to validate their use in further analyses.

Other methods can be used to reveal temporal patterns. Quinn, Halbert & Williams (1991) developed a ‘malleability index’ by regressing the pairwise Euclidean distances between the first three dimensions of ordination space for temporal samples, against environmental variables. ‘Temporal coherence’ measures the degree to which a response variable is similarly variable in time across a set of samples (Code S2; Rusak et al., 1999; Angeler & Johnson, 2012). This method compares the amount of temporal variation in the ordination axis sample scores across a set of samples by using statistics generated by an analysis of variance (ANOVA) of the sample scores (using year and sample as factors) to calculate the ‘intra-class correlation coefficient’. This ranges from −1/(*n −* 1) to +1, where *n* is the number of samples sampled, given that *n* > 1 (for calculation details see Zar (1996) p. 398; Rusak et al., 1999). High positive values of this metric indicate increased coherence of the different samples in composition over time. The measure can be recalculated while dropping each time in sequence (an ordered jackknife) to determine the relative effect of each time on the overall value (Rusak et al., 1999). Different sets of samples or treatments can be compared using a Fisher’s *Z* test (Angeler & Johnson, 2012) or randomisation test in the case of low sample sizes (Rusak et al., 1999). A further method, ‘second-stage multidimensional scaling’ (MDS), results in a second MDS diagram that plots the pairwise similarities between two MDS plots from two different time samples (Clarke et al., 2006). It can therefore be used in combination with permutation tests evaluate the significance of temporal changes (Clarke et al., 2006).

The method of principal response curves (PRC) was first developed by Van den Brink & Ter Braak (1999) for comparing compositional change in treatment samples relative to control samples (Code S2). It is similar to redundancy analysis (RDA) in that a multivariate regression is performed on the sample × taxon matrix constrained by variables of interest, but PRC is explicitly constrained by time and treatment (Lepš & Šmilauer, 2003; Ter Braak & Šmilauer, 2015). PRC uses a single axis, plotted against time, to express trajectories of compositional change relative to a reference treatment/control (Lepš & Šmilauer, 2003: 145); further axes can be calculated if they are of interest. Taxon weights also can be calculated and plotted for each PRC to show the change in the relative abundance (e.g. percent cover) of each taxon in the treatments relative to the control. The significance of the PRC is tested using Monte Carlo simulations (Van den Brink & Ter Braak, 1999).

As with RDA, PRC yields a measure of the percent variance explained for the overall taxonomic composition and how much is attributable to each variable (treatment and time). For example Sugiura, Tsuru & Yamaura (2013) used PRC to show that plant invasion in forests on a small island in the north-western Pacific had little effect on the temporal dynamics of invertebrate communities. This method requires a ‘baseline’ or ‘control’ treatment level to be set for comparison, an attribute that many observational datasets will not have. Furthermore, PRC allows for only one ‘treatment’ variable and each analysis focusses on the change in one sample, relative to control samples. However, Auber et al. (2017) have recently generalised this method so that it can be used to analyse compositional change in multiple samples, but this application removes the ability to consider an environmental or treatment effect. Thus, a principal response curve analysis yields a line graph showing the relative change in taxonomic composition for each ‘treatment’ along one compositional gradient relative to a control (Table 2). We present a worked example of this analysis in the Code S2 supplement.

##### Method 1.6 compositional pivot days

Compositional pivot days is a dissimilarity-based method developed by Lellouch et al. (2014). This method is appropriate for situations where we want to test for the occurrence of fast changes in taxonomic composition across one point in time, such as the application of an experimental treatment or the effects of a rapid change in environmental conditions (Table 2). It works by comparing pairwise distance values for pairs of temporal observations taken at the same sample location to identify the time points where rapid shifts in composition occurred. A ‘pivot day’ is a sampled time point that is identified by pairwise dissimilarities being low before and after that time point, but relatively higher across it. Compositional pivot days are identified either visually from the pairwise distance matrix plot, or using clustering (see Lellouch et al. (2014) for details). This method may be a good option for a preliminary identification of thresholds or rapid shifts in community structure but does not provide a statistical test for the significance of the threshold relative to background. The power of this analysis will increase as the number of sampled time points increases relative to the number of pivot days. An example application of this method is provided in the Code S2 supplement.

##### Method 1.7 community trajectory analysis

Community trajectory analysis quantitatively compares the temporal change in multivariate compositional dissimilarity space among samples using geometric calculations (De Cáceres et al., 2019). These comparisons are based on the relative amounts, rates, and directions of sample movement in compositional space, thus, the method allows flexible testing of a range of specific, quantitative hypotheses of change, such as the divergence or convergence of samples over time. The main advantage of this method, and difference with other methods, is that the overall trajectories of pairs of samples are compared rather than individual time points. This method is especially useful for a small number of spatial replicates because the output can be graphical; plots use arrows on an ordination diagram to show how samples have moved in compositional space over time; however, larger numbers of samples could be graphed separately. We do not illustrate this method here because comprehensive R code and explanations are presented in the R package ‘vegclust’ (De Cáceres, Font & Oliva, 2010) and demonstrative examples are given in De Cáceres et al. (2019).

#### Method 2: rank abundance

Rank abundance distributions (RADs, *a.k.a*. ‘rank abundance curves’), can provide relatively sophisticated visualisations of community dynamics, and rank abundances can be used to quantitatively measure the shift in taxon ranks within samples taken at different time points (Table 2; Code S4; Avolio et al., 2015, 2019; Hallett et al., 2016). RADs are plots of the relative abundances (on the *y*-axis) of all species in a community ranked (on the *x*-axis) from highest to lowest. They are closely related to *k*-dominance curves, in which species ranks are shown on the *x*-axis, but cumulative proportional abundance (dominance) is plotted on the *y*-axis (Lambshead, Platt & Shaw, 1983). If samples taken at different times are plotted separately, the set of RADs can illustrate changes in community structure over time. RADs also can be used to explore community dynamics if a dissimilarity measure, such as Bray–Curtis, is calculated for the species’ ranks at each time point. The resulting temporal dissimilarity of the RADs can be explored by plotting rank dissimilarity against time lag or by ordinating rank dissimilarities in the same way that spatial compositional dissimilarities are examined. For example, temporal changes in RAD dissimilarity can illustrate changes in species evenness over time as might be expected if compositional homogenisation and a decline in evenness occurs because of invasion by an increasingly dominant species (Ruhl, 2008). Rank clocks can be used to visualise changes in composition over longer time series than Venn Diagrams (Collins et al., 2008; Hallett et al., 2016). Here, the rank order of abundance of each species is plotted at each time point around a central point to form a circle; no change in rank orders over time would be indicated by concentric circles (Collins et al., 2008). This method is available in the ‘codyn’ package in R (Hallett et al., 2016; Avolio et al., 2019). RADs can also be combined with measures of dissimilarities to understand which taxa are responsible for changes in composition; this and the comprehensive use of RADs for testing hypotheses of community change are available in Avolio et al. (2015).

#### Method 3: time cores and venn diagrams

Some methods are more descriptive than statistical, but are appropriate for testing for specific types of temporal patterns. For example, ‘time cores’ is a method that identifies ‘core’ groups of taxa that remain present over time (Vallès et al., 2014; D’Souza & Hebert, 2018). Others, such as Venn diagrams (Code S2) and measures of compositional overlap, are useful only for very small numbers of temporal comparisons. These methods are useful for assessing hypotheses of taxon replacement while specifically identifying the taxa driving compositional shifts (Code S2; Table 2).

#### Method 4: zeta diversity

Zeta diversity, a measure of similarity, analyses change in community composition among samples in the dataset (Codes S3 and S4). It is calculated by taking the average number of taxa that are shared between samples that are grouped in pairs, triples, quadruples, and so on (Table 2; Hui & McGeoch, 2014; McGeoch et al., 2019). Zeta diversity estimates compositional turnover from multiple (*n* ≥ 2) samples, rather than the pairwise comparisons used to estimate beta diversity. What is obtained by the method is a series of zeta values (similarity) for different orders of zeta: zeta_1_ to zeta_*n*_, where *n* is the total number of time points. Each of these values represents the average of the number of taxa shared by each set of times in the dataset. For example, zeta_2_ (zeta order 2) represents the average number of taxa shared by all possible pairs of samples in the dataset (equivalent to 1—beta diversity or mean similarity), zeta_3_ represents the average number of taxa shared by all possible subsets of three samples, and so on. Zeta_1_ (zeta order 1) represents the average alpha diversity (taxon richness) of all the samples in the dataset.

If there is complete turnover in community composition in time, at some point, zeta_*i*_ (where *i* ≠ 1) will equal zero when there are no taxa shared by a given sized subset of samples. In contrast, if community composition never changes through time, zeta_*n*_ = zeta_1_. Therefore, the rate of decline in zeta with increasing ‘zeta order’ tells us about the rate of change in community composition in time (‘zeta decline’). ‘Zeta decay’ can be considered analogous to time-lag graphs and time-lag regression analyses, which we described earlier for pairwise dissimilarity measures (Table 2). This means it experiences the same issue of few time points at higher lags, and thus higher uncertainty. The temporal scale(s) of community dynamics can be illustrated by plotting the change in sample zeta diversity of a particular zeta order against the temporal lag of samples, meaning that this method is useful for detecting both directional changes and cyclical patterns. For instance, if a multi-year time series in composition is cyclical (e.g. shows seasonality), zeta would seasonally decrease and then increase. These analyses can be done for different sets of samples to make spatial or temporal comparisons and, if necessary, zeta values can be normalised using the gamma diversity (total number of taxa in the dataset) to account for differences in taxon richness across samples (McGeoch et al., 2019). Further, different methods of creating the subsets for calculating zeta can be used, such as nearest neighbours or relative to a time-zero sample (McGeoch et al., 2019). This enables the user to assess taxonomic turnover at different orders of zeta and understand drivers of turnover in rare and common taxa (Latombe, Hui & McGeoch, 2017). This method could be extended to assess temporal turnover at different orders of zeta by adding time as an ‘environmental’ variable. Code and worked examples for the zeta diversity method is provided in the Codes S3 and S4 supplements and comes with its own R package, ‘zetadiv’ (Latombe et al., 2017).

#### Method 5: synchrony

Synchronicity refers to similarities and differences in the temporal dynamics among groups of taxa. A useful measure of this, called ‘synchrony’, was developed by Loreau & De Mazancourt (2008). This standardised value measures how closely changes in taxon abundances within a community track one another in time. It ranges from zero (complete asynchrony of taxon abundances in the community) to one (perfect synchrony; all taxon abundances changing in the same way) and, by exploring different sized ‘time windows’, can be used to detect scales of temporal variation. Overall significance tests of synchronicity in community change can be obtained by performing null model analysis (Pedersen et al., 2017). We provide code and worked example of this method in the Code S4 supplement, which comes with its own R package, ‘synchrony’ (Gouhier & Guichard, 2014).

A similar method (Houlahan et al., 2007) uses the sum of the temporal variances and pairwise covariances of all taxa in the community to distinguish if the community shows ‘compensatory dynamics’ over time. This method results in either a positive, negative or zero community-level covariance value, indicating that taxa are increasing or decreasing in synchrony (positive community covariance), some taxa are increasing, but this is compensated for by decreases in other taxa (negative community covariance), or that no change is evidence (zero community covariance) (Houlahan et al., 2007; Hallett et al., 2016).

#### Method 6: turnover rates

The degree of temporal variation in community composition can be assessed by calculating the turnover rate of the community (a.k.a. ‘temporal turnover’ or ‘species turnover’), which is a measure of the rate of change in taxonomic composition for a sample over time (Code S3; Aguirre et al., 2003). Turnover rates measure the magnitude of compositional change, but do not tell us about the direction of change or the taxa involved in compositional shifts. This method can detect when, and how rapidly, temporal shifts occur in a temporal sequence to detect complex temporal patterns such as perturbations, tipping points and pivot points (Table 2). Code for a worked example of this method can be found in Code S3.

Turnover rates are calculated using combinations of colonisation, immigration, extinction, mortality, recruitment, and survival. One of the commonly used methods is that of Diamond (1969) where the percentage turnover rate, *T*, is calculated as *T* = 100 × (*E* + *C*)/(*S*1 + *S*2), where *E* is the number of taxa that went extinct between two time points, *C* is the number of taxa that colonised between the same two time points, *S*1 is the number of taxa present at time 1 and *S*2 is the number of taxa present at time 2. *T* gives the percent change in taxon identities over the time period and ranges from 0, representing no turnover, to 1, indicating complete turnover. This measure can be calculated only for a single location at two time points; pairwise turnovers must be calculated separately for datasets with multiple time periods or multiple samples. *T* values can be compared through time, at different time intervals, or at different spatial scales (Boulinier et al., 2001; Tworek, 2004). They also can be used in a regression-style analysis to assess the effects of a treatment variable or other explanatory factor on compositional turnover (Aguirre et al., 2003; Tworek, 2004). Using *T* values in regression in this way is similar to the measure of ‘species turnover’ described above, based on pairwise dissimilarities (Baselga, 2010), because it represents the rate of appearance of new species over time, relative to the total number of species.

The ability to use turnover methods also is tied to the temporal extent of the study for the given system and the average lifespan of taxa. For example, complete turnover in prokaryotes can be observed over a period of days (Kara & Shade, 2009), whilst many decades may be required to detect any species turnover in some plant communities (Kardol et al., 2010). However, because it can be standardised by the number of taxa in the community, *T* is a potentially comparable measure of compositional change among different datasets, if they are on similar timescales and encompass communities with a similar distribution of life-histories.

#### Method 7: joint species distribution models

There are a range of regression approaches that simultaneously model individual species’ community dynamics, such as multispecies *N* mixture models and joint species distribution models (Warton et al., 2015). These methods can be used to model explicit hypotheses of community change, with a focus on interactions among taxa (Dorazio, Connor & Askins, 2015; Ovaskainen et al., 2017a, 2017b) and can work for datasets with reasonably high numbers of species (Thorson et al., 2016), but not yet for the very high numbers of taxa often resulting from molecular analyses. Some of these methods allow for useful additions, such as accounting for uncertainty in the detection of taxa, variation in traits and phylogenetic relatedness. In Hierarchical Modelling of Species Communities (HMSC), species-to-species association matrices are able to be estimated at multiple spatial or temporal scales. This enables a researcher to assess if temporal variation in community composition is due to intrinsic or external factors. Modelling is conducted in a Bayesian framework. We do not illustrate this method here because comprehensive R code and explanations are presented in the R package ‘HMSC’ (Tikhonov et al., 2020) and demonstrative examples are given in the literature cited above.

### Step 3: testing for statistical significance

Some of the methods presented in ‘Step 2’ have their own built-in significance tests, often based on randomisations of the community matrix. Regardless of the multivariate analysis selected, most ecologists interested in community dynamics seek a way to determine if the changes that they see over time in the results of those analyses are greater than, or less than, they would expect statistically by chance alone.

Commonly-used randomisation tests include the Mantel test (Legendre & Legendre, 2012) that can be used to compare pairs of dissimilarity matrices computed at different times and to test for significant difference over time. For example, a robust measure of significance in time-lag regression analysis can be determined by running Mantel tests on the dissimilarity matrix and the matrix of pairwise distances among samples in time, with Monte Carlo permutations (Bêche & Resh, 2007; Day, Carrière & Baltzer, 2017). Null models, whereby community matrices are randomised under given criteria (Gotelli & Graves, 1996; Gotelli, 2000, 2001; Gotelli & Entsminger, 2001; Ulrich & Gotelli, 2007, 2010), can be used to test the significance of any observed change in compositional dissimilarity, and can easily be incorporated into analyses. Permutational multivariate analysis of variance (PERMANOVA) is used to test for significant compositional differences among groups of samples, where the assumption of homogeneity of variances among groups is met (PERMANOVA; Anderson, 2001; Legendre & Legendre, 2012). This assumption can be tested using the ‘betadisp’ function in the vegan package in R (Oksanen et al., 2019). A worked example of the use of the PERMANOVA approach is provided in the Code S2 supplement and a lucid description of possible steps in such analyses using several illustrative examples is given in Clarke (1993).

The primary consideration in applying any randomisation test is what assumptions are being made about taxon niches and whether they are valid. If the interest is in testing the effects of interactions among taxa on their co-occurrence, but the randomisation procedure allows taxa to occur in samples where they are unable to occur for other reasons, such as habitat differences, a difference among samples may be falsely detected (an inflated Type I error rate). Thus, if strong inferences are to be made about compositional differences, we must be sure that the differences among the groups of samples that we are testing are the only difference. Similarly, the sample size needs to be high enough to allow the detection of differences among sets of samples for the given level of compositional variability (Type II error rate).

Schaefer, Gido & Smith (2005) introduced a novel method for detecting significant shifts in community composition using randomisations under null models of taxon mean abundances through time (Code S2). Their approach, which uses both compositional distance (pairwise dissimilarities) and the coefficient of variation (CV), could detect significant dynamics in community composition, even where only small amounts of change had occurred. In this method, temporal changes in communities are assessed by using a compositional distance measure that is selected by the user. This quantifies the multivariate distance between the randomly generated communities (null model randomisations) and observed ‘reference’ samples (e.g. ‘Time 0’ samples, compared to randomisations of ‘Time 1’ samples). If the observed distance is greater than under randomisation, then we can infer statistically significant temporal change. Because this can be done in an ordination context, increases and decreases of particular taxa driving the change can be determined from the ordination plots. Limitations of this method are that it requires a relatively large degree of temporal change in the data compared to spatial variation (i.e. more temporal, than spatial, variation) and it is relatively computationally intensive; however, we provide the R code for a worked example (Code S2).

### Step 4: reporting on the analysis workflow and results

Reporting on aspects of the data analysis workflow is a critical step to ensure the results are reproducible, robust, and represent the variation deemed important for the research aim. The number of taxa, samples, and independent replicates in space and time all should be clearly stated in the Methods or Results section (see Hurlbert (1984) for guidance on statistical independence). The dissimilarity measures selected, and the rationale and procedures used to arrive at that decision, should be reported in the Methods along with a citation. Different methods that are compared, for example in the data exploration phase, should be listed in the Methods to provide justification analysis decisions (see McCune & Grace (2002) for comprehensive advice). A wide variety of methods are available for multivariate community data visualisation (Table 3).

### Case studies

Here, we provide four worked examples to demonstrate different types of multivariate analyses to assess patterns and processes in community dynamics. We used open-access datasets to investigate data with different temporal and spatial structures: (1) a single sample measured multiple times in ant communities, (2) a controlled experiment with treatments applied in plant communities, (3) multiple spatial and temporal replicates in plant communities, and (4) a single site with seasonal sampling in bacterial communities. We present a brief description of each dataset and describe the results and interpretation for different analyses. Detailed R code for each case study is provided as supporting information (Codes S1–S4).

#### Case study 1—ants: a single sample measured multiple times (Code S1)

**Figure 1:**
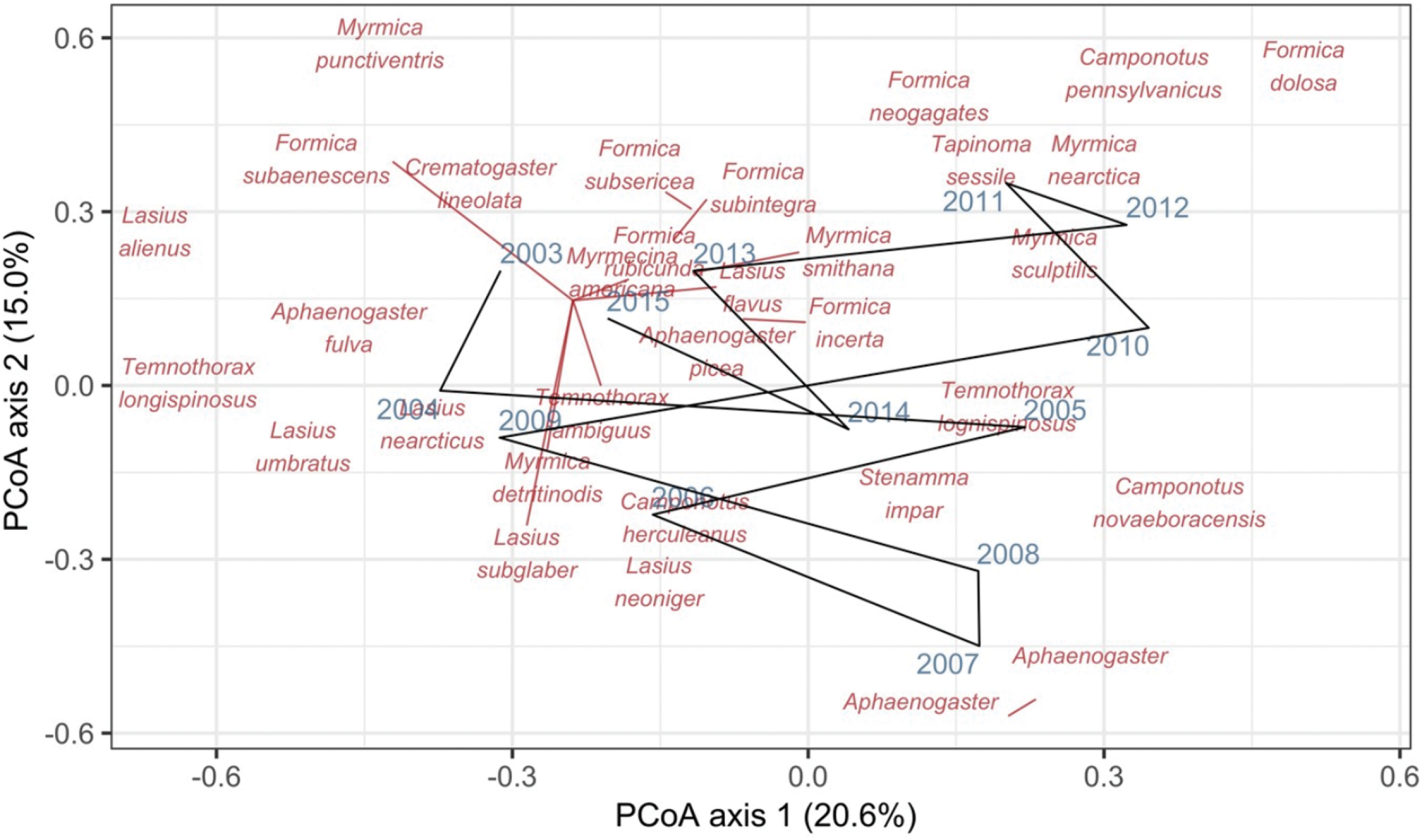
Principal coordinates analysis (PCoA) of Canberra dissimilarities from 13 temporal samples (blue year labels) of ants (red species labels) from a 90 × 90-m forest plot within the Harvard Forest Hemlock Removal Experiment where hemlock was logged in 2005. The black line shows the temporal trajectory linking the yearly samples. Species labels are plotted using coordinates of their correlations with each axis.

**Figure 2:**
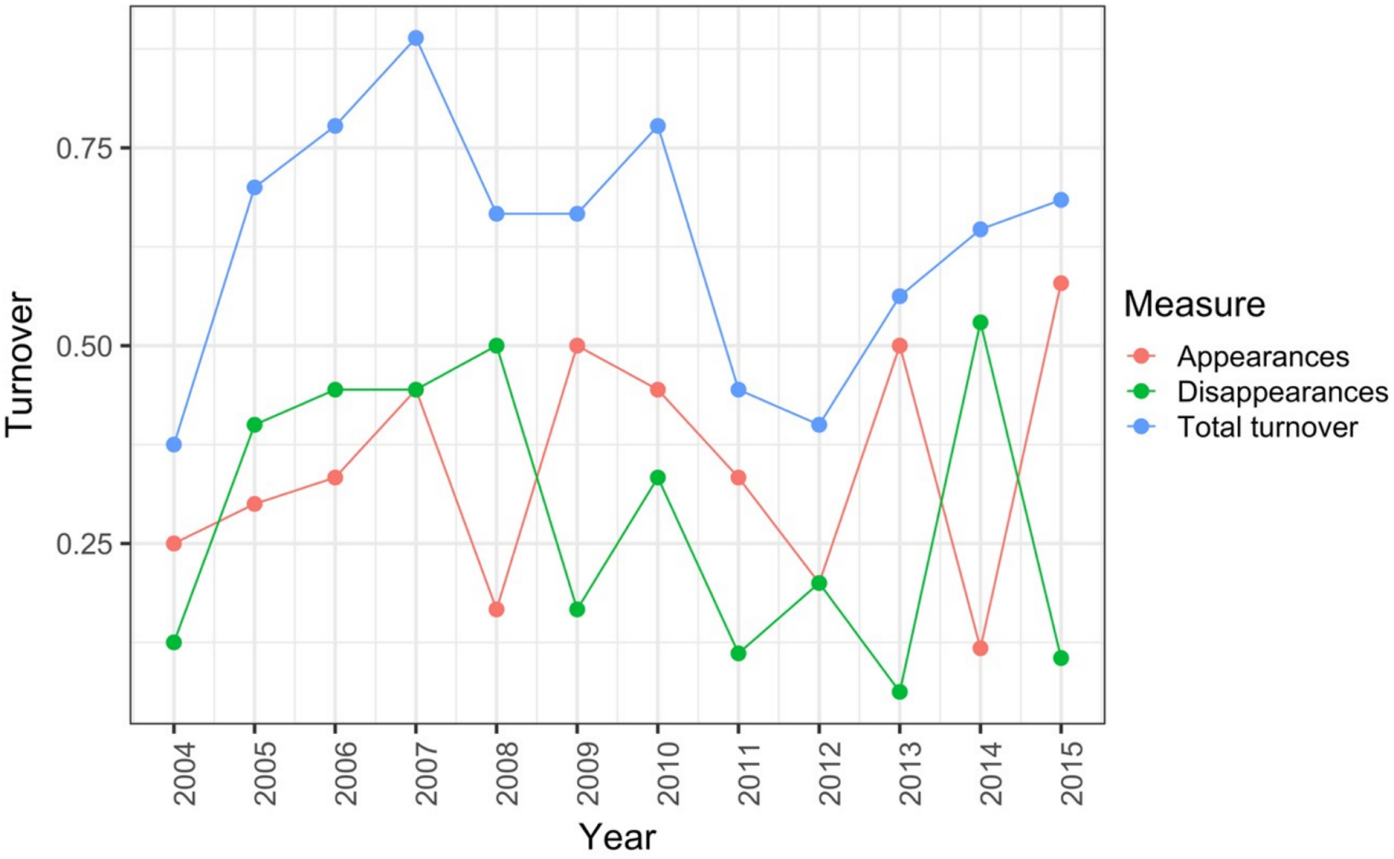
Between-year turnover in ant species composition in a single 90 × 90-m forest plot within the Harvard Forest Hemlock Removal Experiment where hemlock was logged in 2005 (Record et al., 2018). The relative proportion of species appearances, disappearances and their sum (total turnover) are displayed at the endpoint of each between-year period.

**Figure 3:**
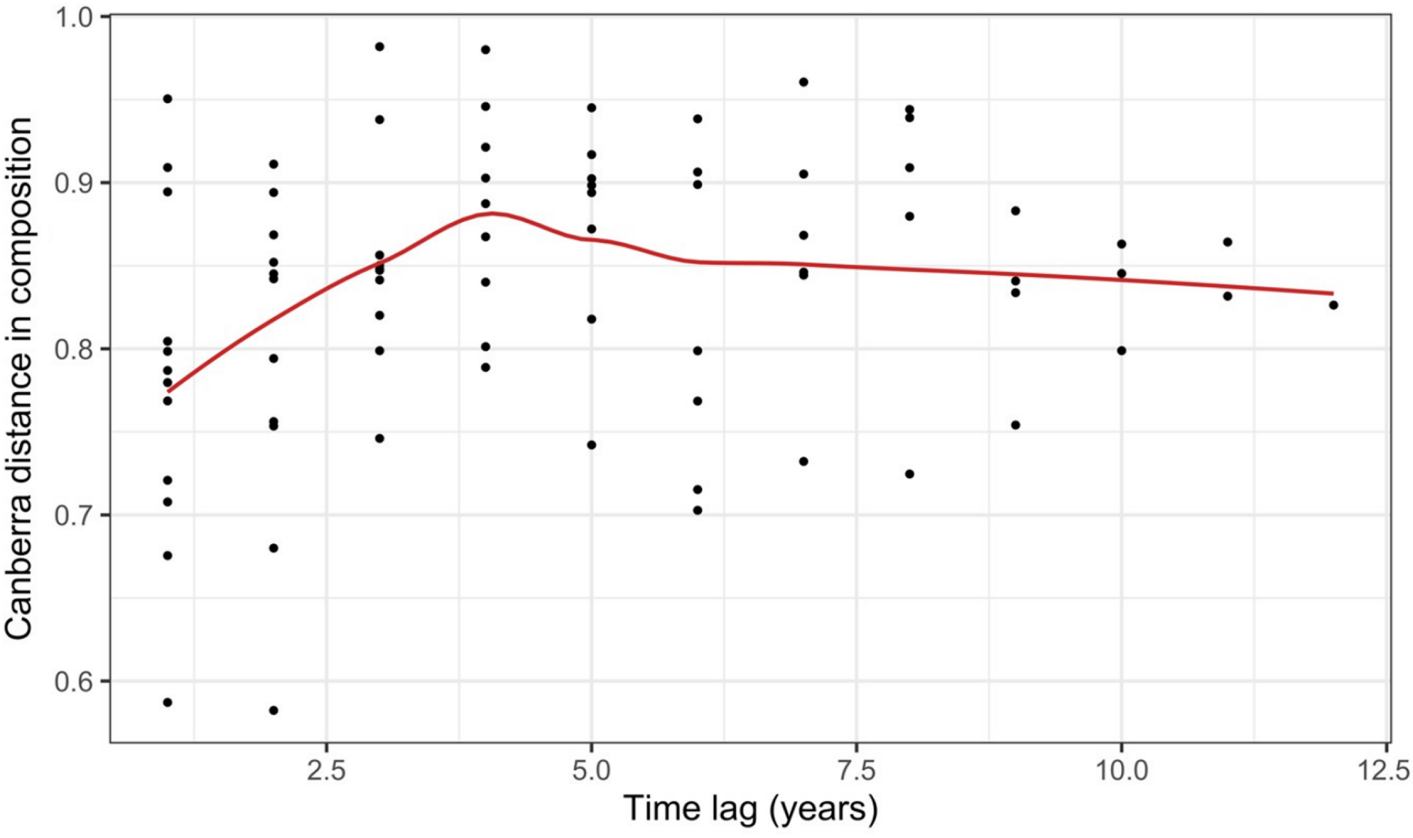
Pairwise temporal dissimilarities calculated using the Canberra metric between yearly ant community samples from a single 90 × 90-m forest plot within the Harvard Forest Hemlock Removal Experiment where hemlock was logged in 2005 (Record et al., 2018). The red line is a loess curve fitted across all points.

Record et al. (2018) measured ant community composition over time in experimentally treated, 90 × 90-m forest plots. We selected one plot, measured annually for 13 years (2003–2015), to demonstrate methods appropriate for detecting compositional change for one sample over time. The focal plot was part of the Harvard Forest Hemlock Removal Experiment (Ellison et al., 2010) whereby *Tsuga canadensis* (eastern hemlock) was removed by logging in 2005 after 2 years (2003–2004) of baseline data collection. An ordination trajectory plot (PCoA based on dissimilarity measure D10, Legendre & Legendre, 2012) showed there was little overall change in composition over the entire sample period (Fig. 1). Turnover analysis showed that compositional changes were driven initially by species losses, followed by species gains (Fig. 2) and the analysis of compositional overlap (Table 1) showed that at the end of the sampling period, the plot contained all the same species as in baseline sampling, along with an additional three species. Time lag analysis showed that the largest compositional differences appeared across approximately 4 years; that is, species composition changed the most at approximately 4-year intervals (Fig. 3).

#### Case study 2—plants: controlled experiment with treatments (Code S2)

Porensky et al. (2016a, 2016b) measured plant species composition annually from 2007 to 2014 in quadrats on 24 transects to which six grazing treatments were applied since 1982 (four transects in each treatment): none (exclosure), light, moderate, heavy, heavy-to-light (lightly grazed since 2007; ‘new light’) and heavy-to-none (new exclosure since 2007). They calculated the mean cover across 25 quadrats for each species on each of the 24 transects at each time (24 samples at each time).

**Figure 4:**
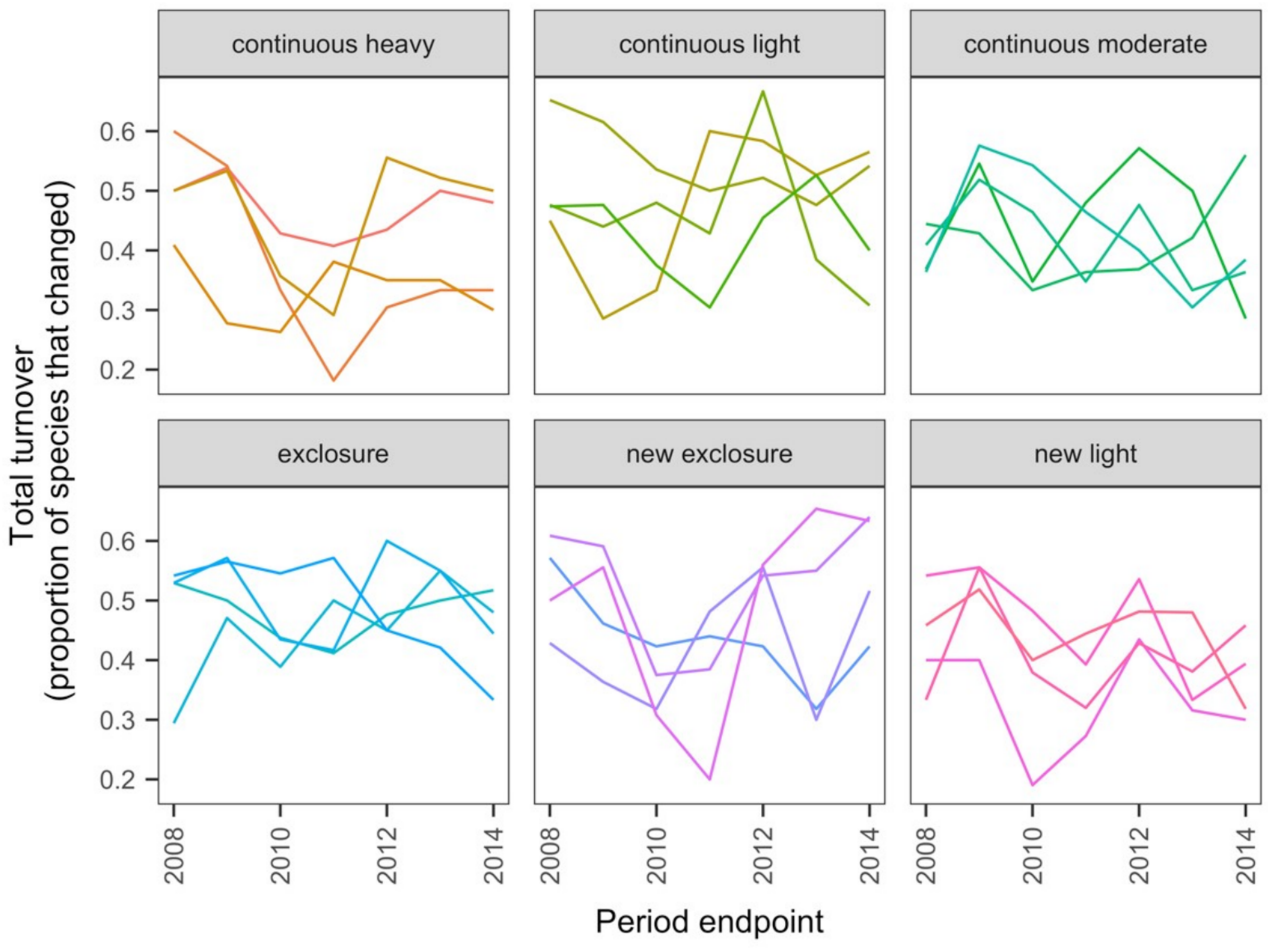
Changes in compositional turnover over time for data from the Porensky et al. (2016a, 2016b) case study. Period endpoints are the final year of the interval over which total turnover in species composition was measured. Twenty-four transects were subjected to one of six grazing treatments and were sampled annually from 2007 to 2014. Grazing treatments were: none (exclosure), light, moderate, heavy, heavy-to-light (lightly grazed since 2007; ‘new light’) and heavy-to-none (new exclosure since 2007). Note that ‘exclosure’ treatment is the control (no grazing). The different lines represent different transects.

**Figure 5:**
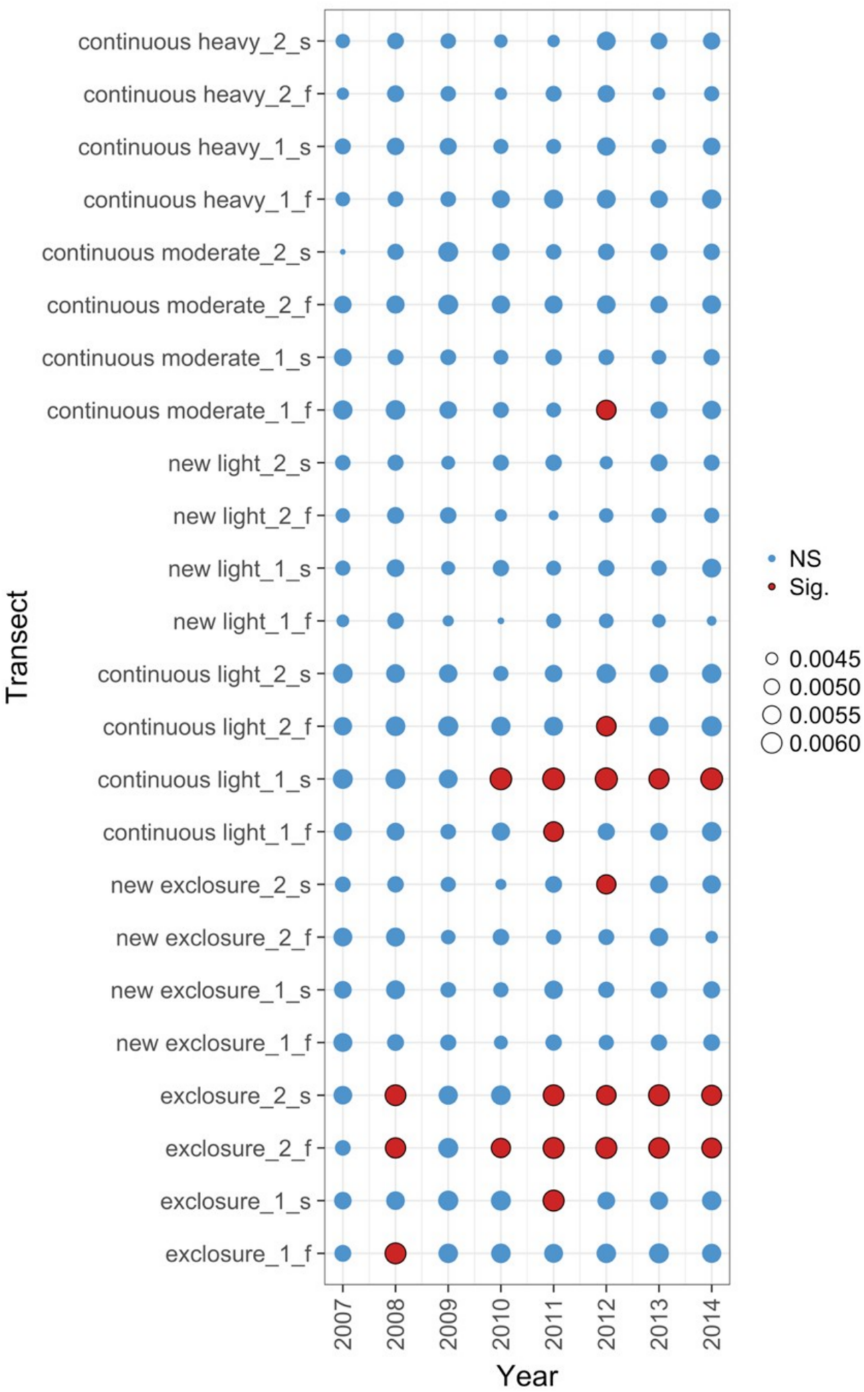
Local contributions to beta diversity (LCBD) for each transect at each sampling time for data from the Porensky et al. (2016a, 2016b) case study. The plot shows the relative contributions of the different transects and times to the overall beta diversity. Symbol size is proportional to the LCBD value and significance is indicated by the red, outlined circles; blue circles are not significant. Transects had a grazing treatment and were established in one of two replicate pastures (1 or 2) and were either north-facing (f) or south-facing (s). Grazing treatments were: none (exclosure), light, moderate, heavy, heavy-to-light (lightly grazed since 2007; ‘new light’) and heavy-to-none (new exclosure since 2007).

**Figure 6:**
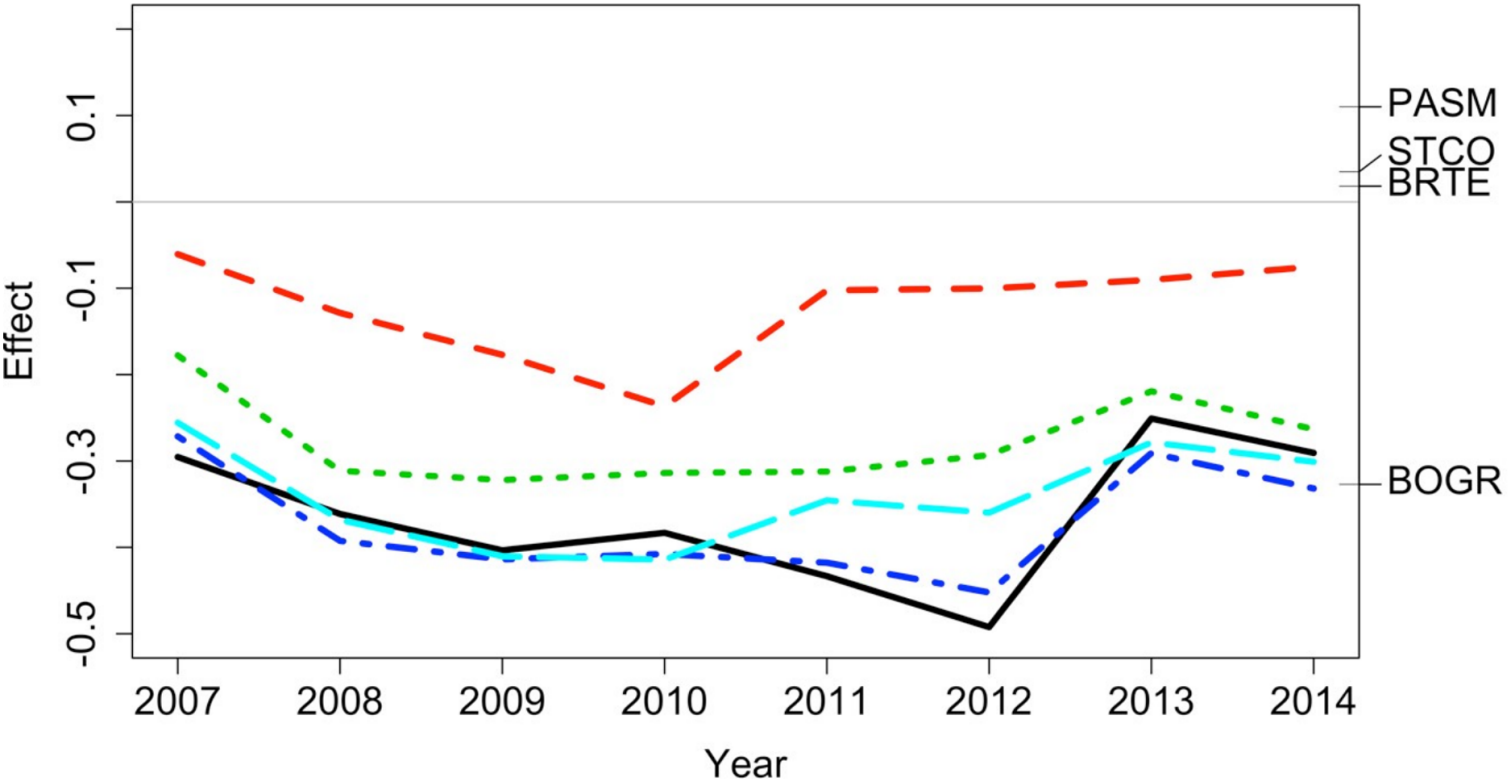
Principal response curve analysis of data from the Porensky et al. (2016a, 2016b) case study. Changes in canonical (regression) coefficients (effect) over time show that treatments differed in plant species composition from the control (exclosure; grey line) but the differences among treatments were largely similar over time. Treatments were: new exclosure (since 2007; dark blue), continuous light (red), new light (since 2007; light blue), continuous moderate (green), and continuous heavy (black). Four particularly influential species where either lower (BOGR = *Bouteloua gracilis*) or higher (PASM = *Pascopyrum smithii*, STCO = *Hesperostipa comata*, and BRTE = *Bromus tectorum*) in abundance than in the control (black horizontal lines represent the abundance of each species relative to the control).

The data exploration phase showed that the 24 transects were dominated across all measurements by ten common species out of a total of 92 species. Species turnover through time was high; mostly 30% or greater for time period-treatment combinations (Fig. 4). PERMANOVA showed that transects were significantly compositionally distinct and changed significantly over time in different ways (*P* < 0.05; Code S1: PERMANOVA outputs; based on dissimilarity measure D10, Legendre & Legendre, 2012). Partitioning spatial and temporal beta diversity showed that two of the control (exclosure) treatments differed significantly (Fig. 5). Principal Response Curves showed that the grazed treatments differed from the control and that the relative abundances of four species in particular were influential in driving these differences among treatments, but did not show increasing divergence over time (Fig. 6). This result is consistent with the analysis of temporal coherence, which showed that transects were not synchronous in their temporal changes (*icc* = −0.04). Thus, we conclude that treatment differences in turnover and shifts in composition were not consistent or directional over time relative to the control.

#### Case study 3—plants: multiple spatial and temporal replicates (Code S3)

**Figure 7:**
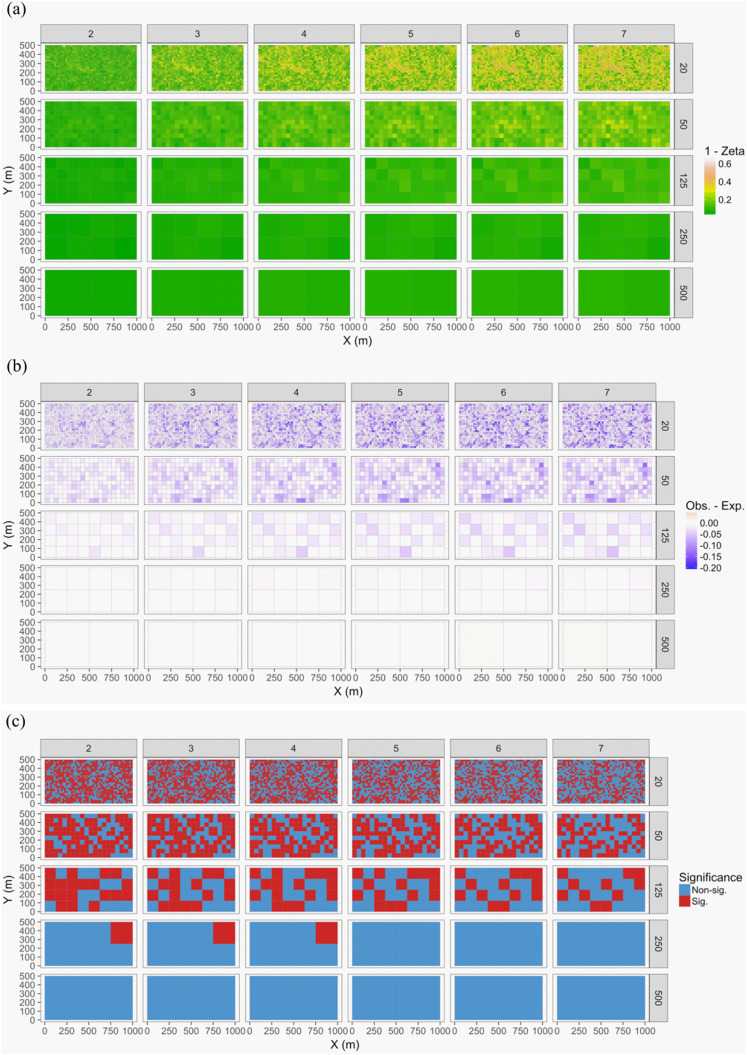
Zeta diversity analysis, based on the number of species shared by a set of temporal samples from the Barro Colorado Island (BCI) case study (Condit, 1998). Results show that the woody forest community in the 50-ha forest census plot on BCI has been significantly more stable over time than random expectation, particularly at large spatial scales where richness is higher. The maps show for 1—zeta diversity calculated for quadrats of increasing sizes (20 × 20, 50 × 50, 125 × 125, 250 × 250 and 500 × 500 m) and for between 2 and 7 orders of zeta (the number of temporal samples compared): (A) the observed values, (B) observed—expected values under a null model of random change in species composition in quadrats over time, and (C) significance of the observed values. Results are given as 1—zeta so that they are comparable to the turnover results in that higher values represent greater dissimilarity in species composition.

The 50-ha forest census plot on Barro Colorado Island (BCI), Panama, has been censused eight times between 1982 and 2015 (Condit, 1998; Hubbell et al., 1999; Hubbell, Condit & Foster, 2005, http://ctfs.si.edu/webatlas/datasets/bci/, 2005). This plot was established in part to test for neutral community dynamics (Hubbell, 1979) by fully censusing all woody stems greater than 1 cm diameter at breast height every five years. The data exploration phase showed there were 324 species generating a highly right-skewed abundance distribution (most species were rare). A line graph of species’ abundance values over time showed very little change in total abundance over time. We found that change in species composition was lower than expected if individual trees were randomly distributed in time (keeping their spatial positions fixed): (1) Zeta diversity analysis showed that the presence of species across census times was more consistent than expected (Fig. 7) and (2) total turnover in species abundances at each of the seven time periods was lower than expected, particularly when the plot data were at larger spatial scales (based on dissimilarity measure D14, Legendre & Legendre, 2012). Thus, we conclude that the observed lack of pattern is consistent with neutral community dynamics; however, when researchers used basal area instead of total abundance in a trajectory analysis, some compositional change after drought was detected (De Cáceres et al., 2019).

#### Case study 4—bacteria: single site with seasonal sampling (Code S4)

**Figure 8:**
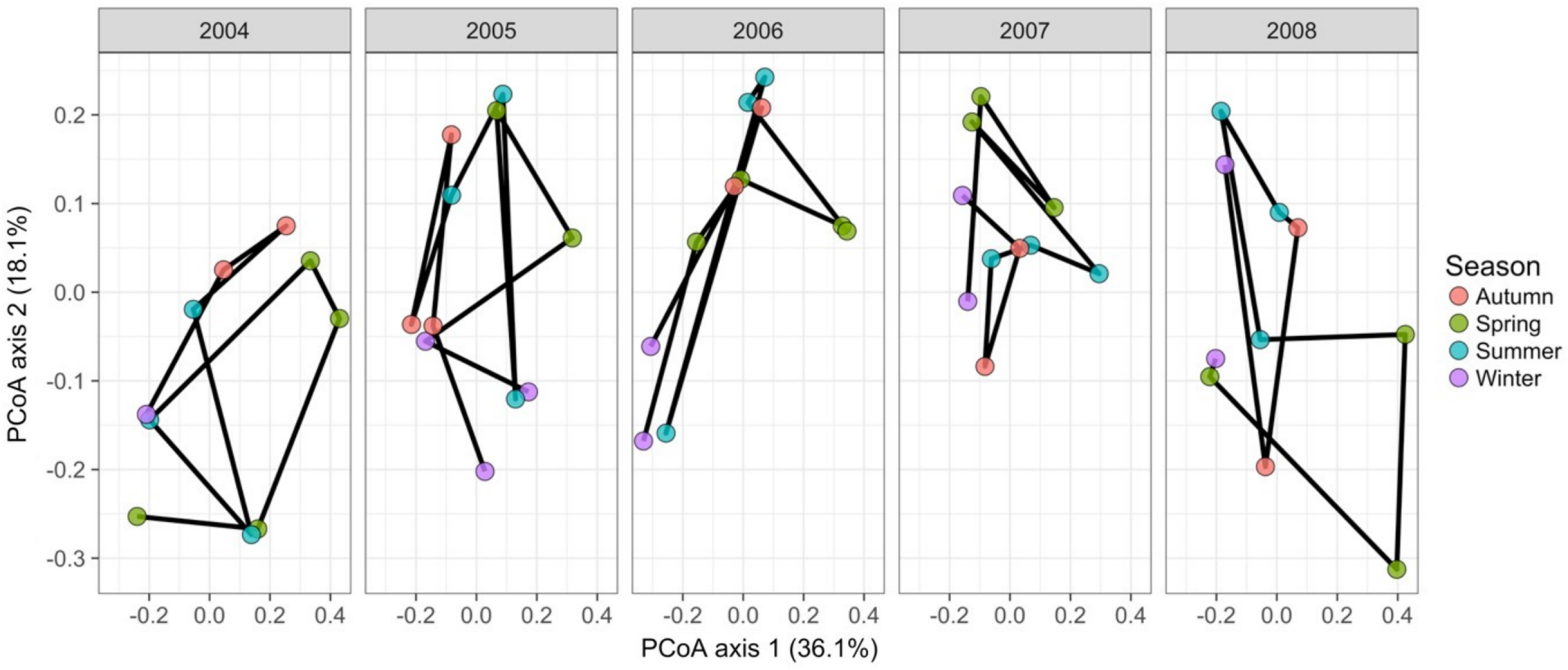
Principal coordinates analysis of 74 marine bacterial OTUs across 62 temporal samples from the Gilbert et al. (2012) case study. Results show no visible consistency in compositional change by season (coloured points) within or between years for the single site analysed. Black lines link samples taken consecutively within years.

Gilbert et al. (2012) measured bacterial taxonomic composition monthly over 6 years using pyrosequencing of the 16S rRNA gene for water samples from a single site off the coast of Plymouth, United Kingdom (source data can be obtained from the journal’s online Supplemental File). The data exploration phase showed that the dataset consisted of 74 operational taxonomic units (OTUs) across 62 temporal samples. Most taxa were common through time. Proportional turnover between temporal samples varied from 4% to 48%, which is relatively low, and was consistent with the detrended correspondence analysis (DCA) result showing a short gradient length of 1.8 for axis 1. Similarly, PCoA graphs showed little consistent change within years (Fig. 8). However, 13 of the OTUs were deemed to be highly variable in that they changed by more than 500 reads across the sampling period (mean number of reads = 296 ± 829 S.D.: row and column summaries, relative abundance distributions and abundance plots).

**Figure 9:**
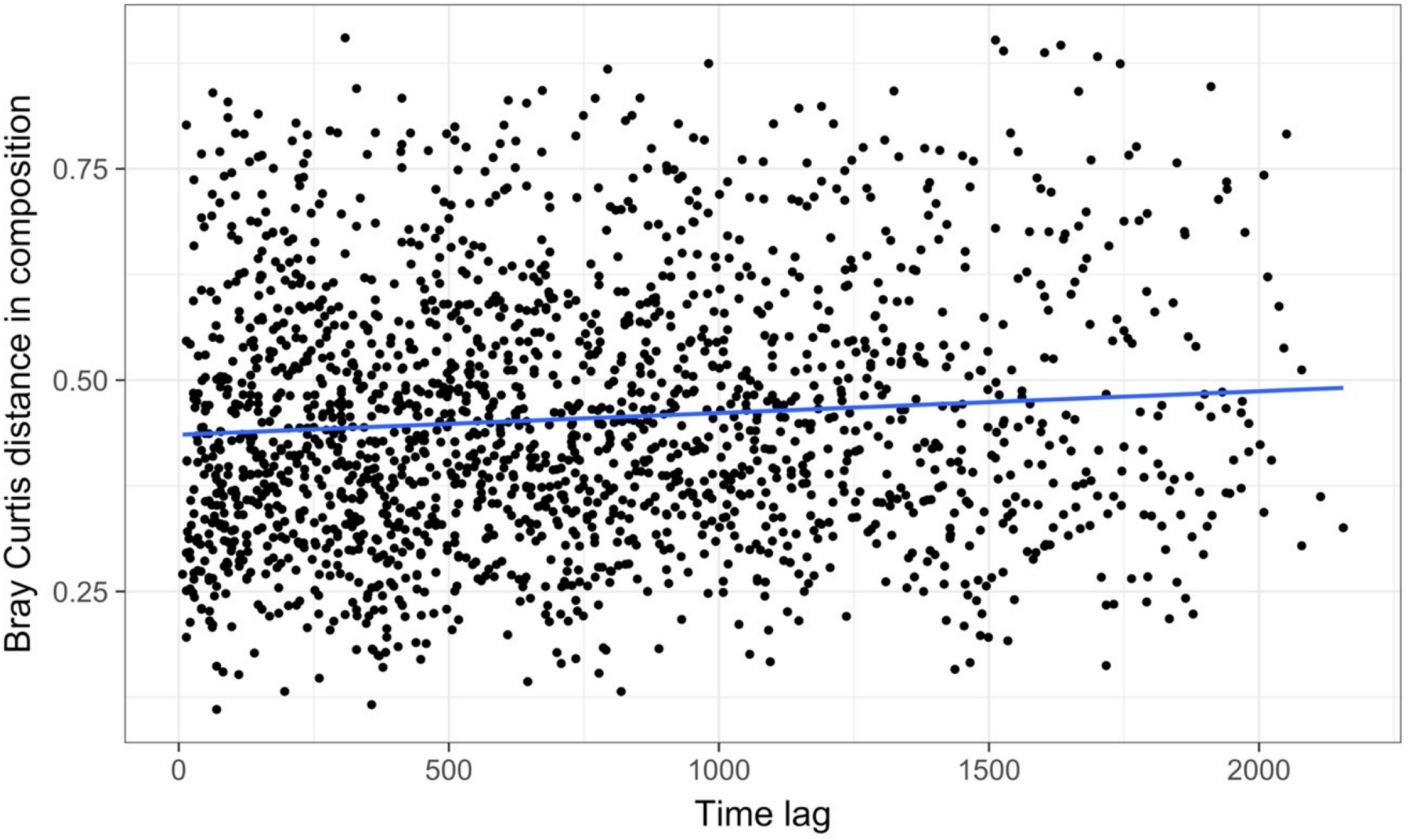
Time lag analysis of 74 marine bacterial OTUs across 62 temporal samples at one site off the coast of Plymouth, United Kingdom from the Gilbert et al. (2012) case study. Results show high variability in pairwise Bray–Curtis dissimilarity values with slight divergence occurring over time. The blue line is a significant linear regression line; however, the high variability suggests that there is not consistent, directional temporal change at this site.

**Figure 10:**
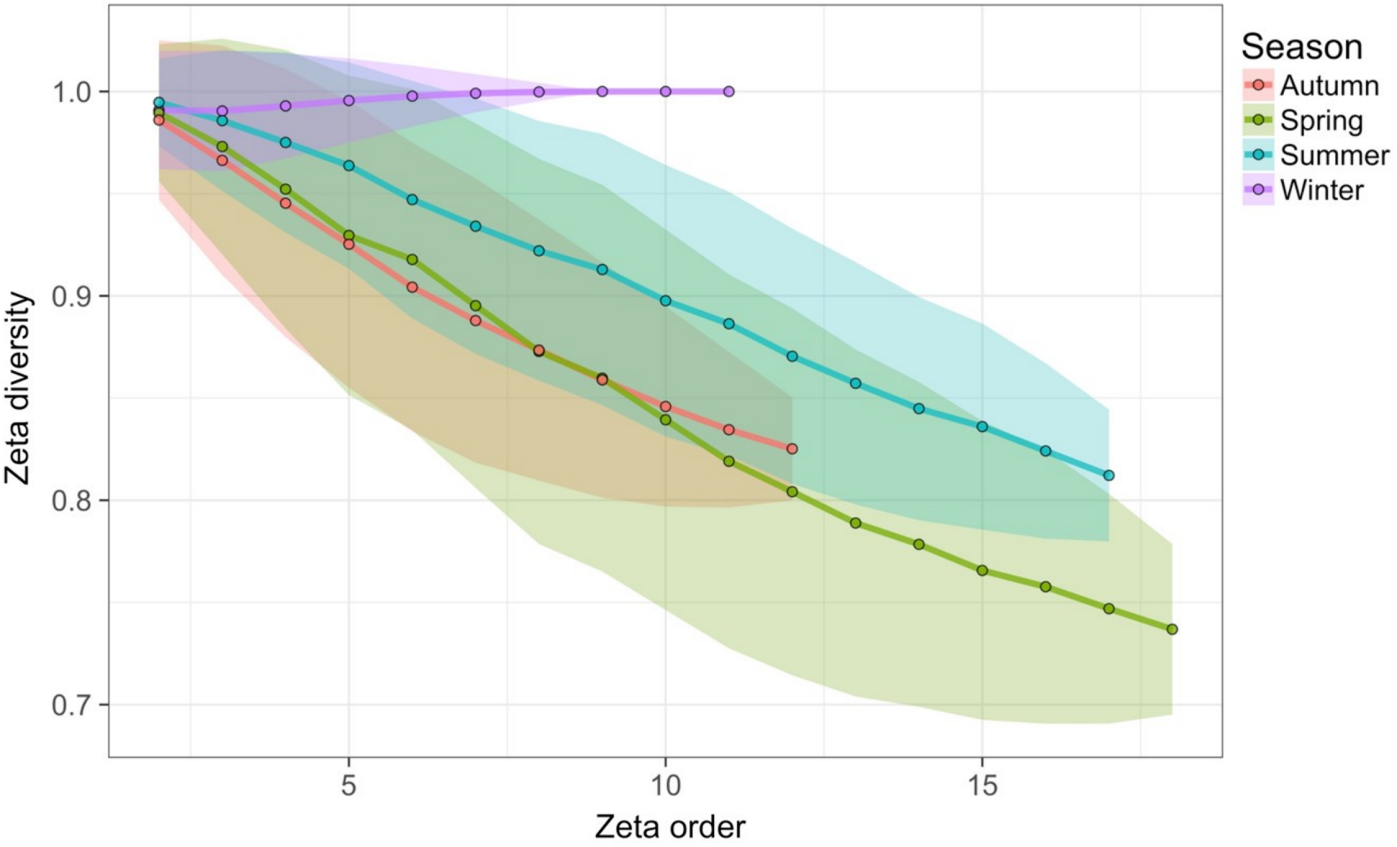
Zeta diversity analysis of the 13 marine bacterial OTUs that were most variable in abundance across 62 temporal samples from the Gilbert et al. (2012) case study. Results show that spring samples were more diverse than winter or summer samples. Zeta order represents the number of temporal samples compared and so each point on the graph shows the mean number of OTUs shared among these different numbers of temporal samples.

**Figure 11:**
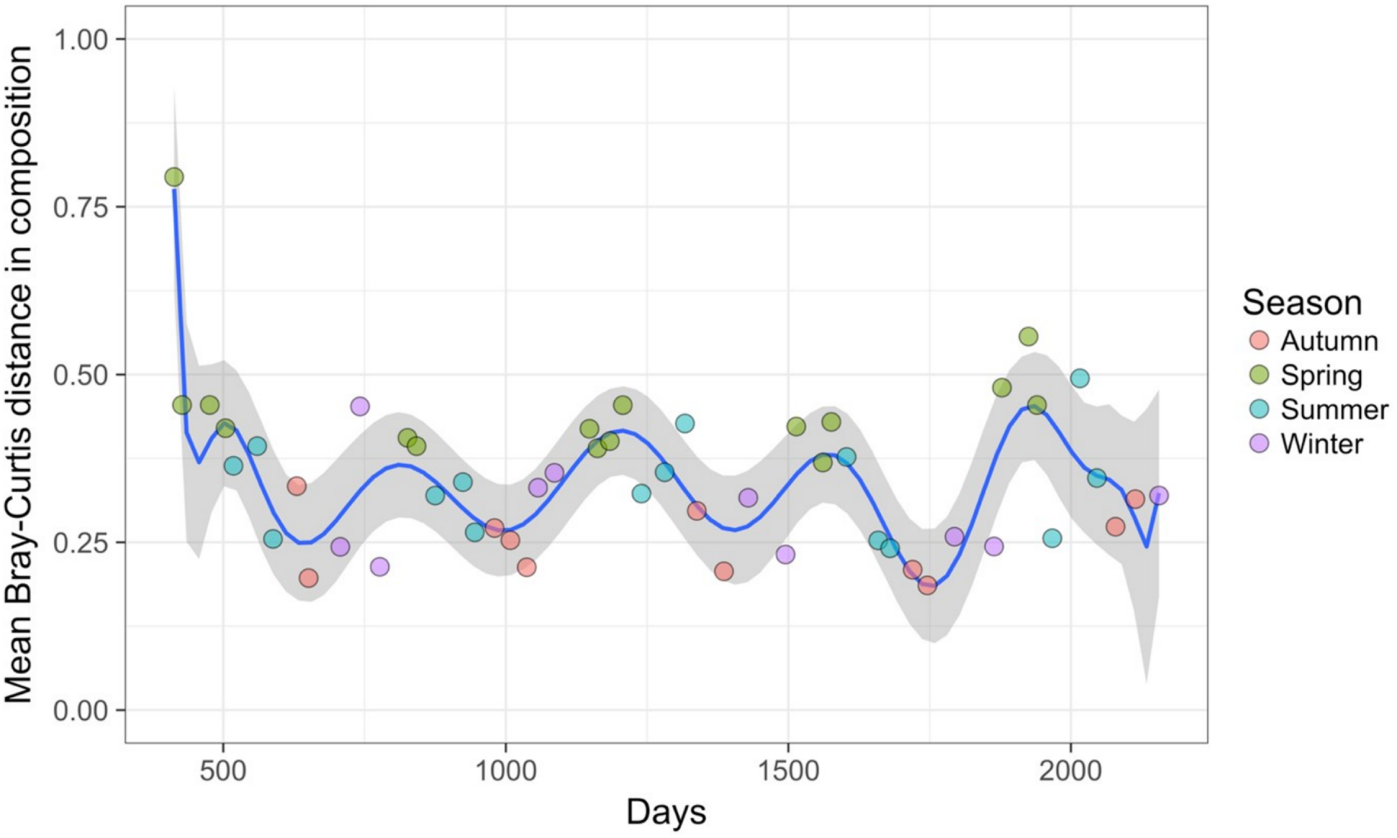
Change in the mean Bray–Curtis dissimilarity values calculated for temporal samples (*n* = 62) grouped by season from the Gilbert et al. (2012) case study. Results are for the 13 most variable marine bacterial OTUs at the one site analysed, showing cyclical community dynamics. The blue line is a polynomial regression line with 16 terms. Multiple comparisons from an analysis of variance showed that samples taken in spring were significantly less similar to other spring samples than were samples taken in summer, autumn, or winter (*P* < 0.001). Samples taken in summer were significantly less similar to other summer samples than were samples taken in autumn (*P* < 0.05).

Time-lag analysis with Bray–Curtis dissimilarities (D14, Legendre & Legendre, 2012) showed that although there was a significant increase in dissimilarity within increasing time lag, variability was high, showing that temporal changes did not occur in a simple divergent or convergent manner (Fig. 9). Temporal synchrony analysis (sensu Loreau & De Mazancourt, 2008) showed that some subsets of OTUs in the community significantly changed in similar ways (Synchrony = 0.27; *P* < 0.05). When patterns in these variable taxa were examined through time using zeta diversity analysis (Hui & McGeoch, 2014), which is based on the presence of species in samples, winter samples shared significantly more species across sets of temporal samples than autumn, spring and summer (Fig. 10; ANOVA, *P* < 0.01). Further analysis of the most variable taxa using the raw dissimilarity values based on abundance data calculated for temporal samples within seasons showed clear cyclical patterns (Fig. 11). Thus, we conclude that the patterns are consistent with seasonal dynamics in community composition, largely driven by a small subset of OTUs that varied in their abundance, rather than presence, over time.

## Discussion

Globally, there is recognition that efforts to collect long-term datasets detailing changes in community structure are lacking, and that such data are useful for understanding the often-complex drivers of community change (Kominoski, Gaiser & Baer, 2018; Kuebbing et al., 2018). Nonetheless, our recent review of thirty years of temporal community dynamics literature (Buckley et al., 2021) shows that the vast majority (75% of reviewed studies) of community change studies are based on time series with relatively few temporal replicates. While this result is not necessarily surprising, given the cost of establishing and maintaining long-term monitoring programmes, we suggest that a considered approach to the choice of available methods for the analysis of temporal community data may reveal nuanced insights into temporal dynamics patterns and their drivers. Here, we outlined a four-step approach to the analysis of multivariate community dynamics data that is an introduction to the complexities of such analyses and can be used to explore options regarding potential analysis methods.

Interactions between community composition and environmental or other factors across space and time are complicated and, while manipulative experiments are useful for understanding such interactions, they are not always possible, especially for long-lived taxa. There are opportunities to apply an ever-expanding set of analyses designed to quantitatively address complex temporal questions using community composition data (Avolio et al., 2019; Legendre, 2019). Nonetheless, there remains a general reliance on well-known descriptive methodologies where community dynamics are assessed via visual outputs of patterns through time (Buckley et al., 2021). The collection of quantitative methods outlined in this present study is an attempt to illustrate the similarities, differences and advantages of the main classes of methods, to assist those wanting to quantitatively assess dynamics, rather than just describe or visualise patterns, focussing particularly on datasets with relatively few temporal replicates.

As ecosystems continue to undergo changes globally, there is an urgent need to generate useful predictions about how communities may continue to respond to ecosystem disturbances, such as climate change, invasive species, and habitat fragmentation (Hobbs, Yates & Mooney, 2007; Kampichler et al., 2012). Ultimately, we hope that the illustrative guide presented here will enable researchers to make more-informed decisions about their choice of analysis methods that will lead to an improved ability to draw strong spatio-temporal inferences from their community datasets.

## Supplemental Information

10.7717/peerj.11096/supp-1 Supplemental Information 1 Example R code for the first worked case study (ant community data).

10.7717/peerj.11096/supp-2 Supplemental Information 2 Example R code for the second worked case study (plant community data).

10.7717/peerj.11096/supp-3 Supplemental Information 3 Example R code for the third worked case study (BCI forest plot dataset).

10.7717/peerj.11096/supp-4 Supplemental Information 4 Example R code for the fourth worked case study (marine bacterial community dataset from high throughput sequencing).

## Additional Information and Declarations

### Data Availability

The following information was supplied regarding data availability: R code is available in the Supplemental Files.
